# Hepatitis C Virus Nonstructural Protein 5A Inhibits Thapsigargin-Induced Apoptosis

**DOI:** 10.1371/journal.pone.0113499

**Published:** 2014-11-19

**Authors:** Xia Jiang, Tatsuo Kanda, Shuang Wu, Shingo Nakamoto, Takaji Wakita, Hiroshi Shirasawa, Osamu Yokosuka

**Affiliations:** 1 Department of Gastroenterology and Nephrology, Chiba University, Graduate School of Medicine, Chiba, Japan; 2 Department of Molecular Virology, Chiba University, Graduate School of Medicine, Chiba, Japan; 3 Department of Virology II, National Institute of Infectious Diseases, Tokyo, Japan; Saint Louis University, United States of America

## Abstract

**Background:**

We previously reported that the hepatitis C virus (HCV) nonstructural protein 5A (NS5A) down-regulates TLR4 signaling and lipopolysaccharide-induced apoptosis of hepatocytes. There have been several reports regarding the association between HCV infection and endoplasmic reticulum (ER) stress. Here, we examined the regulation of HCV NS5A on the apoptosis of hepatocytes induced by thapsigargin, an inducer of ER stress.

**Methods:**

The apoptotic response to thapsigargin and the expression of molecules involved in human hepatocyte apoptotic pathways were examined in the presence or absence of HCV NS5A expression.

**Results:**

HCV JFH1 infection induced ER stress in the Huh7 cell line. HCV NS5A protected HepG2 cells against thapsigargin-induced apoptosis, the effect of which was linked to the enhanced expression of the 78-kDa glucose-regulated protein/immunoglobulin heavy-chain binding protein (GRP78). Consistent with a conferred pro-survival advantage, HCV NS5A reduced poly(adenosine diphosphate-ribose) polymerase cleavage and activation of caspases-3, -7 and -9, and Bax expression, while increasing the expressions of the anti-apoptotic molecules XIAP and c-FLIP. HCV NS5A weakly interacts with GRP78 and enhances GRP78 expression in hepatocytes.

**Conclusion:**

HCV NS5A enhances GRP78 expression, resulting in the inhibition of apoptotic properties, and inhibits thapsigargin-induced apoptotic pathways in human hepatocytes, suggesting that disruption of ER stress-mediated apoptosis may have a role in the pathogenesis of HCV infection. Thus, HCV NS5A might engender the survival of HCV-infected hepatocytes contributing to the establishment of persistent infection.

## Introduction

Hepatitis C virus (HCV) infection is the major cause of hepatocellular carcinoma (HCC) and end-stage liver diseases in the US [1] and Japan [2]. HCV has a positive-strand RNA genome, approximately 9.6 kb in length, which belongs to the *Flaviviridae* family and contains a single open reading frame flanked by 5′ and 3′ untranslated regions (UTRs) [3]. HCV encodes at least 10 structural and nonstructural viral proteins (core, E1, E2, p7, NS2, NS3, NS4A, NS4B, NS5A and NS5B). At least 6 HCV genotypes and more than 50 subgenotypes have been reported based on HCV genomic sequence variation [4].

It has been reported that HCV NS5A plays important roles in viral replication and hepatocarcinogenesis [5]–[7]. Due to these roles, HCV NS5A is an attractive antiviral target and, in fact, HCV NS5A inhibitors are now in clinical use. The combination of HCV NS5A inhibitors and other direct-acting antiviral agents targeting other regions of HCV are a powerful tool for “difficult-to-treat” HCV-infected patients [8]–[10]. HCV NS5A includes an interferon sensitivity determining region (ISDR: NS5A amino acid residues 2209–2248), in which sequence variation is associated with the efficacy of interferon-including treatments for HCV genotype 1b [11]–[13]. Mutations in HCV NS5A ISDR were reported to be associated with a favorable antiviral response and outcome [11].

Endoplasmic reticulum (ER) stress and unfolded protein accumulation in the ER triggers intracellular signaling pathways collectively referred to as the unfolded protein response (UPR) [14]. The activation of UPR enables hepatocytes to either resolve stress or initiate apoptosis [14]–[17]. Our previous study showed that overexpression of the 78-kDa glucose-regulated protein/immunoglobulin heavy-chain binding protein (GRP78/Bip), known to confer resistance to apoptosis, prevented hepatocytes from lipopolysaccharide (LPS)-induced apoptosis [7], [18]. UPR also contributes to hepatic cell damage via the innate immune response [18]. GRP78 plays a role in ER stress pathways and promotes cell survival during UPR [19], [20].

Many cells, including hepatocytes, are programmed to die by apoptosis during mammalian development and when affected by diseases, including liver disease [21]. Failure to undergo apoptosis could result in the accumulation of abnormal cells, leading to hepatitis, cancer and autoimmune diseases [22]. The apoptotic program is initiated by intrinsic stimuli through the mitochondrial release of cytochrome c upon cellular stress, or can be triggered by extrinsic stimuli involving the activation of cell surface receptors such as Fas and the tumor necrosis factor (TNF) receptor [23]. Death-inducing signaling triggers the activation of effector caspases such as caspase-8 and -9 for intrinsic apoptotic pathways, which in turn result in the activation of executor caspase-3, -6 and -7 [24], [25]. Caspase activation during apoptosis induces morphological and physiological cellular changes through the cleavage of poly (ADP-ribose)polymerase (PARP), endonucleases and proteases, leading to cell death [26]. Apoptosis also involves the modulation of B-cell lymphoma-2 (Bcl-2) family proteins, balancing the anti-apoptotic members: Bcl-2, B-cell lymphoma-extra large (Bcl-xl), cellular FADD-like interleukin-1beta-converting enzyme (FLICE)-like inhibitory protein (c-FLIP) and X-linked inhibitor of apoptosis protein (XIAP); with the pro-apoptotic members: Bcl-2-associated X protein (Bax) and tumor protein p53 (p53), as well as regulation of mitochondrial alterations [23].

Several studies have reported the association between HCV and ER stress [27]. It has also been reported that several HCV structural (core, E1 and E2) and nonstructural proteins (NS2 and NS4B) could induce ER stress [28]–[31]. Induction of both ER and oxidative stress by HCV proteins may contribute to the promotion of hepatocyte growth, as well as the inhibition of apoptosis in hepatocytes [3], [7]. These phenomena might contribute to HCV replication in the face of innate and adaptive immunity, leading to hepatocarcinogenesis. In the present study, we examined whether HCV infection induced ER stress in hepatocytes. We also focused on HCV NS5A protein and examined its effect on ER stress-induced apoptosis.

## Materials and Methods

### Cell Culture

Human hepatoma cell lines HepG2 and Huh7 were cultured at 37°C in Dulbecco's modified Eagle's medium (Invitrogen, Carlsbad, CA, USA) containing 10% heat-inactivated fetal bovine serum, 100 units/ml penicillin and 100 µg/ml streptomycin (Sigma, St. Louis, MO, USA) under 5% CO_2_. Huh7 cells were infected with HCV genotype 2 JFH1 [32] as previously described [33]. The stable cell lines HepG2 control expressing pCXN2 [34], and HepG2-NS5A, which expressed pCXN2-HCV genotype 1b NS5A, have been described previously [7].

### Reagents and Plasmids

Antibody against HCV NS5A was purchased from Meridan Life Science, Inc. (Memphis, TN, USA). Bax, Bcl-2, caspase-3, -7, -9, GRP78, c-FLIP and PARP were obtained from Cell Signaling Technology (Danvers, MA, USA). Antibodies targeting Bcl-xL, X-linked inhibitor of apoptosis protein (XIAP) and GAPDH were obtained from Santa Cruz Biotechnology (Santa Cruz, CA, USA). Anti-tubulin was purchased from Abcam (Cambridge, MA, USA). Thapsigargin was purchased from Biovision (Milpitas, CA, USA). The mammalian cell expression plasmid pCXN2 was kindly provided by Prof. Miyazaki J, Osaka University [34]. In the present study, we also used a pCXN2-NS5A expression vector containing an HCV genotype 1b NS5A, including 2 wide-type, 2 intermediate-type, or 1 mutant-type interferon sensitivity determining region (ISDR, aa2209-aa2248), referred to as pCXN2-NS5A-W1 and pCXN2-NS5A-W2, pCXN2-NS5A-I1 and pCXN2-NS5A-I2, or pCXN2-NS5A-M1, respectively. HCV cDNA stocks were obtained from sera in our laboratory, which were collected for other studies after written consent was obtained from all patients. Further explanation of this study was provided on the notice board in our university hospital, and we informed patients that they could withdraw from study participation by their own will at anytime. This study was approved by the Ethics Committee of Chiba University School of Medicine, No. 1462 and 1753. pCXN2-NS5A-I1 was previously described as pCXN2-HCV genotype 1b NS5A [7]. The plasmids pFLAG/CMV2 (pFLAG) and pFLAG-human GRP78 (pFLAG-GRP78) were kindly provided by Prof. Kim WU, Catholic University of Korea, Seoul, South Korea [35]. An ER stress response element (ERSE)-directed luciferase reporter construct (pERSE-luc) was purchased from Qiagen (Hilden, Germany) [36].

### Western Blot Analysis

Western blotting was performed as previously described [7]. Briefly, cell lysates were collected in sodium dodecyl sulfate sample buffer. After sonication for 5 min, protein samples were subjected to electrophoresis on 5–20% polyacrylamide gels and transferred onto polyvinylidene difluoride membranes (ATTO, Tokyo, Japan). Membranes were probed with specific antibodies as indicated. After washing, membranes were incubated with secondary horse-radish peroxidase-conjugated antibodies. Signals were detected by means of enhanced chemiluminescence (GE Healthcare Japan, Tokyo, Japan) and scanned by image analyzer LAS-4000 and Image Gauge (version 3.1) (Fuji Film, Tokyo, Japan). Band intensities were determined by ImageJ software [37].

### RNA Purification, Real-time RT-PCR and Human Apoptosis PCR Array

Cellular RNA was extracted using the RNeasy Mini Kit (Qiagen). One microgram of RNA was reverse-transcribed with the PrimeScript RT reagent (Perfect Real Time; Takara, Otsu, Japan). PCR amplification was performed on cDNA templates using primers specific for GRP78, X-box binding protein 1 (XBP1), DNA damage-inducible protein 34 (GADD34), C/EBP homologous protein (CHOP) and glyceraldehyde-3-phosphate dehydrogenase (GAPDH) [36]. For RNA quantification, real-time PCR was performed using the Power SYBR Green Master Mix (Applied Biosystems, Forester City, CA, USA) according to the manufacturer's protocol. Data analysis was performed based on the ddCt method.

Human apoptosis real-time RT-PCR arrays were performed according to the manufacturer's protocol (Qiagen). The data were analyzed by PCR Array Data Analysis Software (http://www.sabiosciences.com/pcrarraydataanalysis.php).

### siRNA Transfection

HepG2-NS5A cells were transfected with 20 nM of siRNA for GRP78 (siRNA-GRP78) [36] or a negative control siRNA (Santa Cruz) as si-control [36] using Effectene transfection reagents (Qiagen) according to the manufacturer's protocol.

### Transfection and Luciferase Assay

Approximately 1×10^5^ HepG2 cells were placed in 6-well tissue culture plates (Iwaki Glass, Tokyo, Japan) 24 h prior to transfection. Transfection of 0.1 µg of pERSE-luc and 0.1 µg of pCXN2 or pCXN2- NS5A into the cells was performed using Effectene transfection reagents (Qiagen). Cells were treated with or without 0.1 µM thapsigargin for 24 h. Luciferase activity was measured with a luminometer (Luminescencer-JNR II AB-2300, ATTO).

### Crystal Violet Assay

HepG2 control or HepG2-NS5A cells were plated in 6-well plates, and 24 h later, cells were incubated with 0–1 µM thapsigargin. After 48 h of treatment, cells were fixed for 30 min with methanol and stained for 30 min with 0.1% crystal violet [7].

### Measurement of Caspase-3/7 Activities

The Caspase-Glo 3/7 assay (Promega, Madison, WI, USA) was used to determine caspase-3 and -7 activities according to the manufacturer's instructions. Briefly, 1×10^4^ cells were seeded onto 96-well white plates (MS-8096W, Sumitomo Bakelite, Tokyo, Japan). After 24 h, 0.5 µM thapsigargin was added and incubated for 6 h in 5% CO_2_ at 37°C. Caspase-Glo 3/7 reagent was added at a 1∶1 ratio with the medium containing cells in each well of the 96-well plates, and left for 0.5 h at room temperature. Luminescence was recorded as a function of caspase-3 and -7 activities using the Luminescencer-JNR II AB-2300 (ATTO). Medium without cells was used as nonspecific background. The ratios of caspase-3 and -7 activities from each group relative to untreated control groups, defined as 1, were determined by luminescence.

### Apoptosis Assay

HepG2 control cells were transfected with 0.3 µg of pFLAG/CMV2 (pFLAG) or pFLAG-human GRP78 (pFLAG-GRP78) vector (kindly provided by Prof. Kim WU) using Effectene transfection reagents (Qiagen) according to the manufacturer's protocol. At 48 h post-transfection, cells were treated with 0.1 µM thapsigargin for 24 h, and then the APOPercentage Apoptosis Assay (Biocolor, Belfast, Northern Ireland) was used to quantify apoptosis according to the manufacturer's instructions [36]. Transfer and exposure of phosphatidylserine to the exterior surface of the membrane has been linked to the onset of apoptosis. Phosphatidylserine transmembrane movement results in the uptake of the APOPercentage dye by those cells undergoing apoptosis. Purple-red stained cells were identified as apoptotic cells by light microscopy. The number of purple-red cells/300 cells was counted as previously described [36].

### Immunofluorescence

Cells were washed and fixed with 3.7% formaldehyde, followed by blocking with 3% horse serum albumin. Cells were incubated with an HCV NS5A-specific monoclonal antibody and a GRP78 antibody (Santa Cruz) for 1 h. Cells were washed and incubated with anti-mouse immunoglobulin secondary antibody conjugated with Alexa Fluor 488 or anti-rabbit immunoglobulin secondary antibody conjugated with Alexa Fluor 555 (Cell Signaling) for 1 h at room temperature. Nuclear staining was performed with Hoechst 33342, trihydrochloride, trihydrate (Molecular Probes, Eugene, OR, USA). Finally, cells were washed and mounted for confocal microscopy (ECLIPSE TE 2000-U, Nikon, Tokyo, Japan), and the images were superimposed digitally to allow for fine comparisons [33].

### Nucleotide sequence accession number

All sequence reads have been deposited in the DNA Data Bank of Japan (DDBJ) under accession numbers AB983775, AB983776 - AB983779.

### Statistical Analysis

Results are expressed as mean ± standard deviation (SD). Statistical analysis was performed by Student's t-test. A P-value of <0.05 was considered statistically significant. All statistical analyses were performed using DA Stats software (O. Nagata, Nifty Serve: PAF01644).

## Results

### HCV infection induces ER stress in Huh7 cells

**Figure 1:**
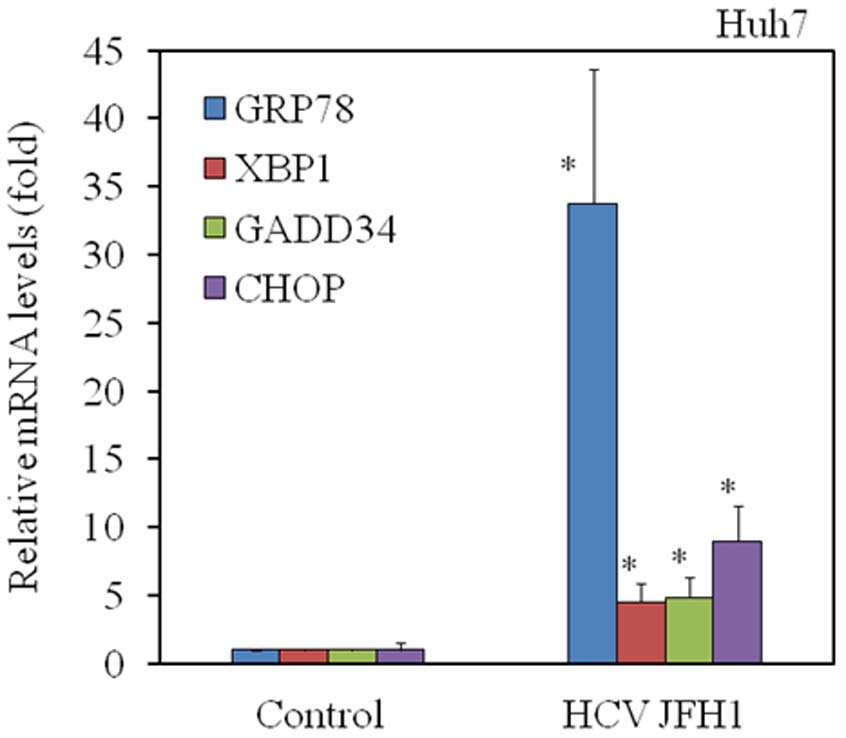
Hepatitis C virus (HCV) infection induces ER stress in hepatocytes. HCV JFH1 infection of Huh7 cells up-regulates mRNA expression of GRP78, XBP1, GADD34 and CHOP. Total cellular RNA was isolated from cells 72 h after infection with HCV. Intracellular gene expression levels of GRP78, XBP1, GADD34, CHOP and GAPDH were measured by real-time RT-PCR. The ratios of GRP78/GAPDH, XBP1/GAPDH, GADD34/GAPDH and CHOP/GAPDH are presented as induction (n-fold) relative to the levels observed in mock-infected control. Data are expressed as mean ± standard deviation. *P<0.05.

To examine whether HCV infection induces the expression of ER stress-associated mRNAs, we infected Huh7 with HCV genotype 2 (clone JFH1) or examined a mock-infected control [7]. Total RNA was isolated at 72 h post-infection, and the message levels of GRP78, XBP1, GADD34 and CHOP were examined by real-time RT-PCR. The levels of GRP78, XBP1, GADD34, and CHOP mRNA expression were enhanced compared to mock-infected controls (Figure 1). Together, our results demonstrated that HCV infection in Huh7 cells up-regulates the mRNA expression of ER stress-associated molecules.

### HCV NS5A protects HepG2 cells from thapsigargin-induced apoptosis

**Figure 2:**
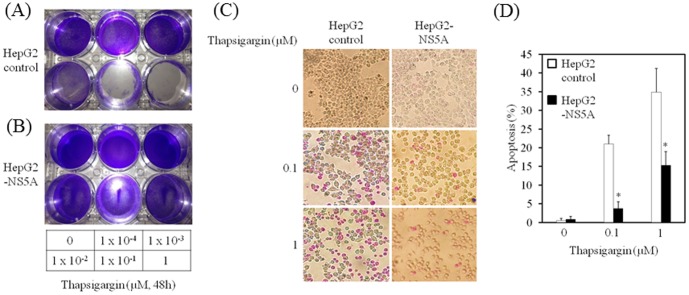
Hepatitis C virus (HCV) nonstructural protein 5A (NS5A) protects hepatocytes from thapsigargin-induced cell death. HepG2 control (A) and HepG2-NS5A (B) cell lines were cultured for 48 h with thapsigargin at 0, 1×10^−4^, 1×10^−3^, 1×10^−2^, 1×10^−1^, and 1 µM. Cells were washed and stained with crystal violet. (C), (D) HCV NS5A protects hepatocytes from thapsigargin-induced apoptosis. HepG2 control and HepG2-NS5A cells were cultured for 24 h with thapsigargin at 0, 1×10^−1^, and 1 µM. Apoptosis was evaluated using the APOPercentage Apoptosis Assay. Purple-red stained cells were identified as apoptotic cells by light microscopy. The number of purple-red cells/300 cells was counted. Data are expressed as mean ± standard deviation. *P<0.05.

Previously, we have reported that HCV NS5A inhibits the LPS-mediated apoptosis of hepatocytes [7]. Thapsigargin, a known ER stress-inducer, specifically inhibits ER Ca^2+^-ATPase, transiently increasing the level of cytosolic free calcium and subsequently inducing apoptosis in human hepatoma cells [38]. To examine the effects of HCV NS5A on apoptosis induced by thapsigargin, we treated the HepG2-NS5A and HepG2 control cell lines with a range (0–1 µM) of thapsigargin and examined them for indications of cell death after 48 h (Figure 2A and 2B). Treatment with 0.1–1 µM thapsigargin induced massive cell death in HepG2 control, but not in HepG2-NS5A cells. Quantification of apoptosis demonstrated a significant increase in apoptotic cell death in HepG2 control, compared with HepG2-NS5A cells after 24 h of treatment with 0.1–1 µM thapsigargin (Figure 2C and 2D).

### HCV NS5A protects HepG2 cells from thapsigargin-induced PARP-cleavage

**Figure 3:**
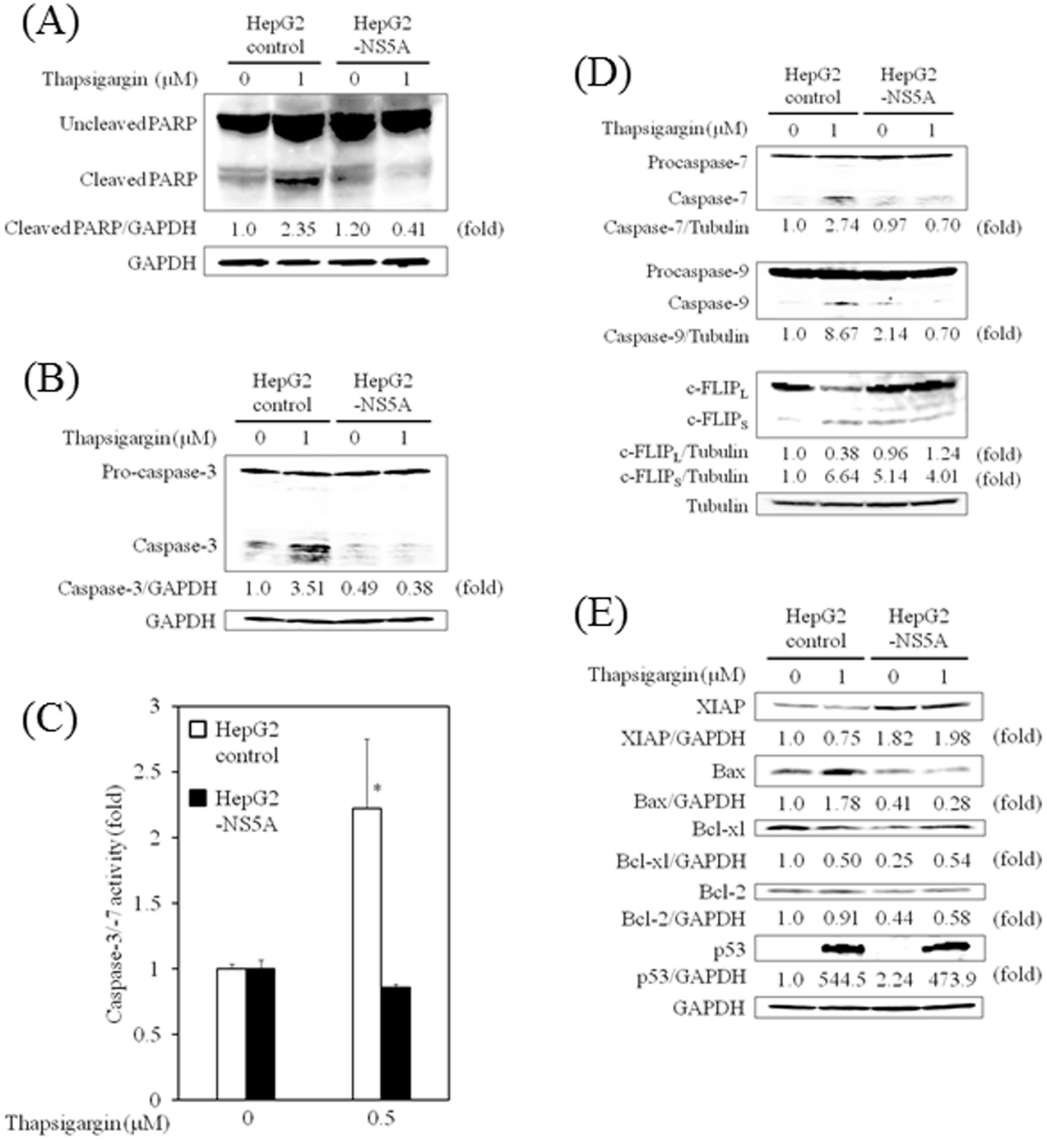
Hepatitis C virus (HCV) nonstructural protein 5A (NS5A) protects hepatocytes from thapsigargin-induced apoptosis. (A) HCV NS5A inhibits PARP cleavage in HepG2 cells. Western blot analysis shows the expression of PARP and cleaved PARP in HepG2 control and HepG2-NS5A cells treated for 24 h with or without thapsigargin (1 µM). Blots were reprobed with GAPDH-specific antibodies to assess equivalent protein loading. (B) HCV NS5A inhibits caspase-3 expression in HepG2 cells. Western blot analysis shows the expression of procaspase-3 and caspase-3 in HepG2 control and HepG2-NS5A cells treated for 24 h with or without thapsigargin (1 µM). (C) HCV NS5A inhibits the caspase-3/-7 activity in HepG2 cells. The Caspase-Glo 3/7 assay (Promega, Madison, WI, USA) shows the caspase-3/-7 activity in HepG2 control and HepG2-NS5A cells treated for 6 h with or without thapsigargin (0.5 µM). Data are expressed as mean ± standard deviation. *P<0.05. (D) HCV NS5A inhibits caspase-7/-9 and enhances cellular FADD-like interleukin-1beta-converting enzyme (FLICE)-like inhibitory protein, long form (c-FLIP_L_) expression in HepG2 cells. Western blot analysis shows the expression of procaspase-7 and caspase-7 (upper panel), procaspase-9 and caspase-9 (middle panel) and c-FLIP_L_ and c-FLIP, short form (c-FLIP_S_), in HepG2 control and HepG2-NS5A cells treated for 24 h with or without thapsigargin (1 µM). Blots were reprobed with tubulin-specific antibodies to assess equivalent protein loading. (E) HCV NS5A enhances XIAP expression and inhibits Bax expression in HepG2 cells. Western blot analysis shows the expression of XIAP, Bax, Bcl-xl, Bcl-2 and p53 in HepG2 control and HepG2-NS5A cells treated for 24 h with or without thapsigargin (1 µM). Blots were reprobed with GAPDH-specific antibodies to assess equivalent protein loading. Densitometric analyses were performed using ImageJ software.

Considering that HCV NS5A promoted the survival of thapsigargin-treated HepG2 cells (Figure 2), we investigated whether HCV NS5A interfered with apoptosis using Western blot analysis to detect PARP cleavage and the expression of mature caspase-3, -7 and -9, and c-FLIP, as well as measuring caspase-3/-7 activities. PARP cleavage, induced by 1 µM thapsigargin, was observed in HepG2 control cells but was barely detectable in HepG2-NS5A cells (Figure 3A). Procaspase-3 cleavage by 1 µM thapsigargin treatment was also observed in HepG2 control cells but was barely detectable in HepG2-NS5A cells (Figure 3B). As shown in Figure 3C, the caspase-3/-7 activities measured in the presence of 0.5 µM thapsigargin were increased in HepG2 control cells as compared with HepG2-NS5A cells. We also observed procaspase-7 cleavage as well as procaspase-9 cleavage in HepG2 control cells but they were again barely detectable in HepG2-NS5A cells 24 h post-treatment with 1 µM thapsigargin (Figure 3D).

### HCV NS5A up-regulates the expression of c-FLIP and XIAP and down-regulates the expression of Bax in thapsigargin-induced apoptosis

c-FLIP comes in two forms, FLIP short (FLIP_S_) and FLIP long (FLIP_L_) [39]. We observed less c-FLIP_L_ in HepG2 control cells, compared to HepG2-NS5A after 24 h of treatment with 1 µM thapsigargin (Figure 3D). On the other hand, the level of c-FLIPs increased to a lesser extent in thapsigargin-treated HepG2 control cells (Figure 3D), although we observed no differences in procaspase-8 cleavage between HepG2 control and HepG2-NS5A cell lines (data not shown). Interestingly, there have been several reports describing c-FLIP as having an anti-apoptosis role [39], [40].

We observed that the expression of XIAP, an anti-apoptotic molecule and inhibitor of caspase-3/-7/-9 [41], was increased in HepG2-NS5A cells as compared with HepG2 control cells after 24 h of treatment with 1 µM thapsigargin (Figure 3E). We also observed that the expression of Bax, a pro-apoptotic molecule, was decreased in HepG2-NS5A cells as compared with HepG2 control cells after 24 h of treatment with 1 µM thapsigargin (Figure 3E). PCR array analyses also demonstrated that XIAP mRNA and Bcl-2 mRNA were up-regulated approximately 1.87-fold and 1.83-fold, respectively, in HepG2-NS5A cells, as compared to HepG2 control cells (Table 1, *P<0.05*). Real-time RT-PCR analysis revealed that XIAP mRNA and Bcl-2 mRNA were down-regulated approximately 0.72-fold and 0.81-fold, respectively (*P<0.05*), but Bax mRNA was not up-regulated in HepG2-NS5A transfected with siRNA-GRP78 as compared to HepG2 control cells.

**Table 1:** Differences in induction of anti-apoptotic genes by thapsigargin in HepG2-NS5A cells compared to HepG2 control cells.

Gene name	Fold change*	P-values	Gene title
TNF	10.85	0.022	Tumor necrosis factor
XIAP	1.87	0.0017	X-linked inhibitor of apoptosis
BCL2	1.83	0.018	B-cell chronic lymphocytic leukemia (CLL)/lymphoma 2
IGF1R	1.74	0.00035	Insulin-like growth factor 1 receptor
BRAF	1.72	0.0059	V-Raf murine sarcoma viral oncogene homolog B1
BCL2L1	1.71	0.00023	BCL2-like 1
HRK	1.69	0.011	Harakiri, BCL2 interacting protein [contains only BCL2 homology 3 (BH3) domain]
IL10	1.65	0.16	Interleukin 10
BIRC3	1.61	0.00011	Baculoviral IAP repeat containing 3
CFLAR	1.58	0.00083	Caspase-8 and Fas-associated via death domain (FADD)-like apoptosis regulator
RIPK2	1.57	0.0086	Receptor-interacting serine-threonine kinase 2
BCL2L2	1.54	0.00086	BCL2-like 2
NFKB1	1.23	0.11	Nuclear factor of kappa light polypeptide gene enhancer in B-cells 1
BAG3	1.17	0.016	BCL2-associated athanogene 3
BFAR	1.16	0.014	Bifunctional apoptosis regulator
MCL1	1.08	0.013	Myeloid cell leukemia 1
NAIP	1.05	0.26	Nucleotide binding and oligomerization domain (NOD)-like receptor (NLR) family, apoptosis inhibitory protein

We compared the induction of anti-apoptotic genes by thapsigargin in HepG2-NS5A with that in HepG2 control. Three sets of real-time PCR arrays were performed. *, HepG2-NS5A vs. HepG2 control.

### HCV NS5A up-regulates GRP78 expression in human hepatoma cell lines

**Figure 4:**
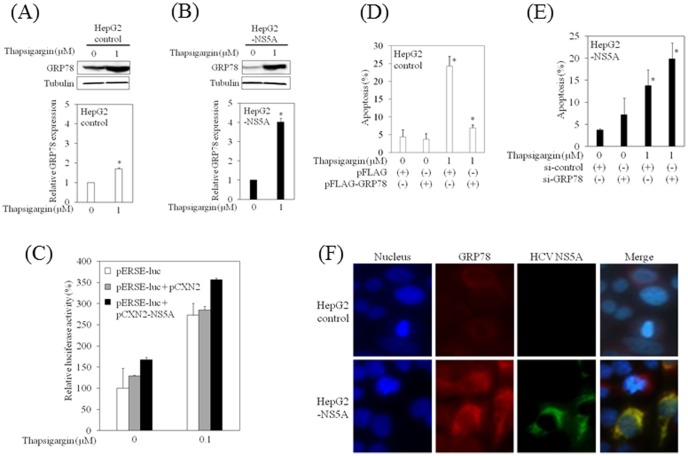
Hepatitis C virus (HCV) nonstructural protein 5A (NS5A) up-regulates GRP78 expression in hepatocytes. (A), (B) HCV NS5A enhances GRP78 expression in HepG2 cells. Western blot analysis shows the expression of GRP78 in HepG2 control (A) and HepG2-NS5A cells (B) treated for 24 h with or without thapsigargin (1 µM). Blots were reprobed with tubulin-specific antibodies to assess equivalent protein loading. Densitometric analyses were performed using ImageJ software. Data are expressed as mean ± standard deviation. *P<0.05. (C) HepG2 cells were transiently transfected with pCXN2 or pCXN2-NS5A and an ER stress response element (ERSE)-directed luciferase reporter construct (pERSE-luc). Luciferase assays were performed 48 h after transfection. Data are expressed as mean ± standard deviation of triplicate determinations from 1 experiment representative of 3 independent experiments. (D) The effects of overexpression of GRP78 on thapsigargin-induced apoptosis in HepG2 control cells. Apoptosis was evaluated using the APOPercentage Apoptosis Assay. Purple-red stained cells were identified as apoptotic cells using light microscopy. The number of purple-red cells/300 cells was counted. Data are expressed as mean ± standard deviation. *P<0.05. (E) The effects of GRP78 knockdown on thapsigargin-induced apoptosis in HepG2-NS5A cells. (F) HCV NS5A specifically co-localizes with GRP78. HepG2 cells were transiently co-transfected with 0.1 µg pCXN2 or pCXN2-NS5A. HCV NS5A expression was probed for an anti-HCV NS5A primary antibody and Alexa-Fluor-488 secondary antibody. Endogenous GRP78 was detected by an anti-GRP78 primary antibody and Alexa-Fluor-555 secondary antibody.

We expected the association between the induction of GRP78 and the anti-apoptotic actions of HCV NS5A in thapsigargin-induced apoptosis. To further analyze the mechanism at work, we examined the expression of GRP78, an anti-apoptotic protein, by Western blotting (Figure 4A and 4B). We confirmed that GRP78 was induced by thapsigargin in HepG2-NS5A cells at elevated levels as compared to HepG2 control (4-fold vs. 1.8-fold, compared to the respective baseline levels). We performed a reporter assay to elucidate the molecular mechanisms by which HCV NS5A increased GRP78 expression in hepatocytes (Figure 4C). Transient transfection of HepG2 cells with the pCXN2-NS5A expression vector induced ERSE reporter activity.

We also examined the effects of GRP78 expression on thapsigargin-induced apoptosis in HepG2 control cells, observing that overexpression of FLAG-GRP78 rescued HepG2 control cells from apoptosis (Figure 4D). In contrast, knockdown of GRP78 enhanced thapsigargin-induced apoptosis in HepG2-NS5A cells (Figure 4E).

To compare the localization of endogenous GRP78 with that of HCV NS5A, HepG2 cells were transfected with pCXN2-NS5A or pCXN2 alone. After 48 h, cells were stained with a mouse monoclonal HCV NS5A antibody and rabbit polyclonal GRP78 antibody. Confocal microscopy revealed co-localization of GRP78 with HCV NS5A (Figure 4F). We also tested whether HCV NS5A interacts with GRP78 by co-immunoprecipitation, but no co-immunoprecipitations of GRP78 and HCV NS5A were detected under our experimental conditions (data not shown). Together, our data suggest that HCV NS5A might weakly interact with GRP78, resulting in enhanced GRP78 expression in hepatocytes. Nevertheless, further studies will be needed.

### Mutations in HCV NS5A ISDR have no impact on thapsigargin-induced apoptosis in hepatocytes

**Figure 5:**
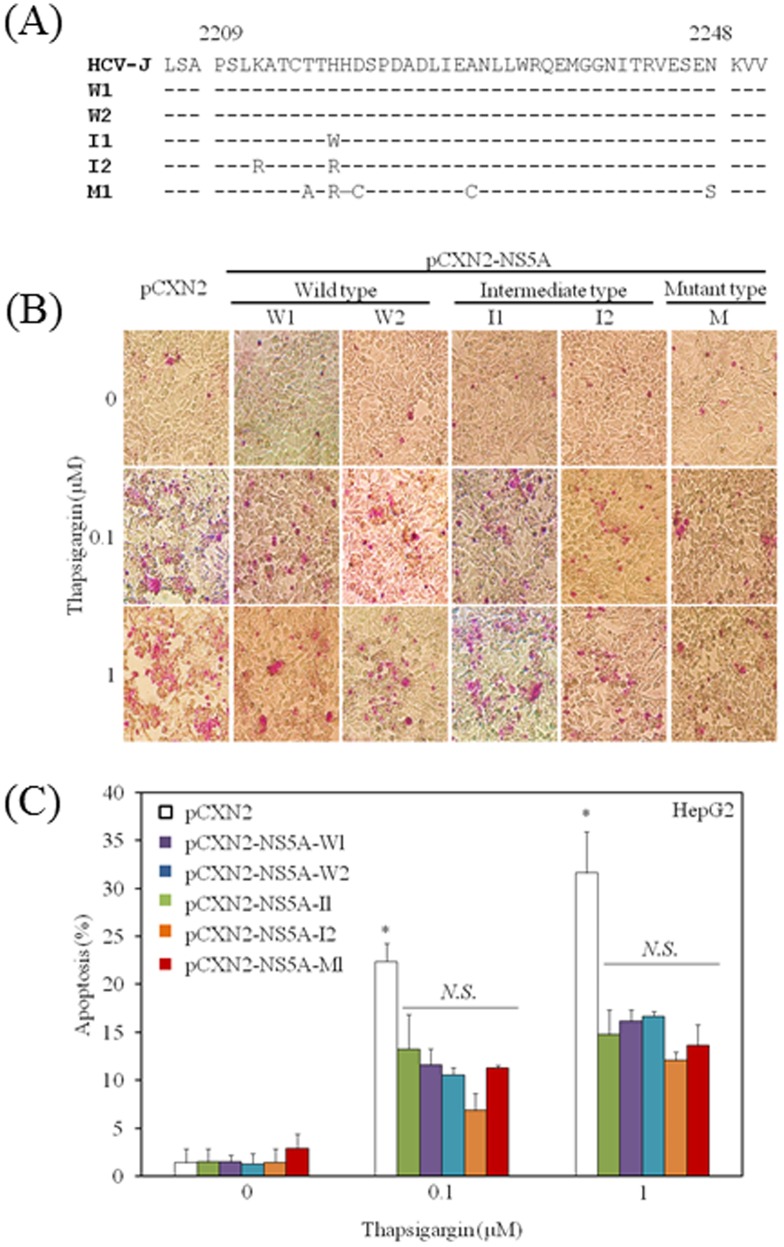
Mutations in the hepatitis C virus (HCV) nonstructural protein 5A (NS5A) interferon-sensitivity determining region (ISDR) do not have impact on thapsigargin-induced apoptosis in hepatocytes. (A) Amino acid sequences of the HCV NS5A ISDRs of pCXN2-NS5A in the present study. W, wild type; I, intermediate type; M, mutant type. The sequence of HCV-J was reported by Kato et al. [42]. (B), (C) No effect of HCV NS5A ISDR sequences on apoptosis was observed by thapsigargin. HepG2 cells were transfected with 0.3 µg of each vector as indicated. 24 h post-transfection cells were treated with thapsigargin at the indicated concentrations. Apoptosis was evaluated at 48 h post-transfection by APOPercentage Apoptosis Assay. Purple-red stained cells were identified as apoptotic cells by light microscopy. The number of purple-red cells/300 cells was counted. Data are expressed as mean ± standard deviation. *P<0.05.

It is known that HCV NS5A ISDR has an impact on the treatment response in HCV genotype 1b-infected patients receiving interferon-including regimens [11]. Enomoto et al. [11] analyzed 84 patients with chronic HCV genotype 1b infection who had received interferon alpha for 6 months, observing that a sustained virological response did not occur in any of the 30 patients whose NS5A2209-2248 sequences were identical to that of HCV-J (wild type); 5 of 38 patients with 1 to 3 amino acid changes in the NS5A2209-2248 region (intermediate type) had a complete response, as did all 16 patients with 4 to 11 changes in NS5A2209-2248 (mutant type) [11]. As shown in Figure 5A, we constructed HCV NS5A-expression plasmids containing the wild type, intermediate type or mutant type ISDR sequences from cDNA stocks in our laboratory.

Compared with the HCV-J sequence [42], 2, 2 and 1 plasmids have wild-type, intermediate-type and mutant-type ISDR sequences, respectively. Next, we examined thapsigargin-induced apoptosis after transient transfection with each ISDR expression vector. Briefly, HepG2 cells were transiently transfected with each vector. From 24 h post-transfection, cells were treated with thapsigargin for 24 h and apoptosis was evaluated (Figure 5B and 5C). No impact of the HCV NS5A ISDR sequences on thapsigargin-induced apoptosis was observed in the present study. These results might suggest the absence of correlation between the ISDR domain of HCV NS5A and ER stress.

## Discussion

We demonstrated that HCV infection induced ER stress in hepatocytes, supporting a previous report [27]. However, it remains unclear how HCV can avoid ER stress-mediated apoptosis in infected hepatocytes. The increase of GRP78 expression by HCV NS5A might play a critical role in the negative regulation of ER stress-induced apoptosis in hepatocytes, although we did not examine whether other HCV proteins up-regulate GRP78 expression. This process negatively regulates the anti-apoptotic effects of GRP78, including caspase activation and PARP cleavage, presumably to counteract the deleterious effects of thapsigargin on hepatocyte viability.

Naturally, HCV replicates in hepatocytes, leading to chronic hepatitis, cirrhosis and HCC [1], [2]. Upregulation of GRP78 is one of the mechanisms preventing the apoptosis of HCV-infected hepatocytes induced by ER stress, which supports previous observations that GRP78 is activated in HCC tissues [36], [43]. HCV NS5A might also induce UPR in order for HCV to survive. In fact, resistance to ER stress-induced apoptosis in infected cells might play a critical role in HCV replication or HCV-related pathogenesis. Although HCV NS5A ISDR has been shown to have a critical impact on the interferon response in HCV genotype 1b patients [11], mutations of HCV NS5A ISDR had no effects on thapsigargin-induced apoptosis here. It seems that there is no correlation among the ISDR domain of HCV NS5A, anti-apoptotic functions and ER stress.

It has been shown that HCV NS5A engages in the ER-nucleus signal transduction pathway [44], [45]. HCV NS5A causes the disturbance of intracellular calcium and the resultant Ca^2+^ signaling triggers the elevation of reactive oxygen species in mitochondria, leading to the translocation of nuclear factor kappa B (NF-κB) and signal transducer and activator of transcription 3 (STAT3) into the nucleus [44]. We demonstrated that HCV NS5A induced the activation of ESRE promoter activity (Figure 4C) and also observed the interaction between HCV NS5A and GRP78 (Figure 4F), supporting the previous studies [44], [45].

On the other hand, HCV NS5A impairs TNF-mediated hepatocyte apoptosis [46] and LPS-induced hepatocyte apoptosis [7]. Thapsigargin, one of the ER stress-inducers, transiently increases the level of cytosolic free calcium and subsequently induces hepatocyte apoptosis [38]. In the present study, we observed that HCV NS5A inhibits thapsigargin-mediated hepatocyte apoptosis.

Christen et al. reported that the activation of the ER stress response by hepatitis viruses up-regulates protein phosphatase 2A, which is involved in many important cellular processes including cell-cycle regulation, apoptosis, cell morphology, development, signal transduction and translation [47]. Noxa is a Bcl-2 homology domain-containing pro-apoptotic mitochondria protein [48]. ER stress augments the expression of Noxa following lytic viral infection [48]. In the present study, we observed that HCV NS5A impaired the ER stress modulation of the pro-apoptotic molecule Bax, and that HCV NS5A impaired the reduction of the anti-apoptotic molecule XIAP by ER stress. It was reported that HCV NS5A is a potential viral Bcl-2 homologue that interacts with Bax and inhibits apoptosis in HCC cells [49]. XIAP is located downstream of NF-κB signaling, which is activated by HCV NS5A through ER stress [44]. In the present study, we also demonstrated the upregulation of c-FLIP by HCV NS5A, supporting the previous observation that HCV NS5A increased the expression of c-FLIP [7].

The ER chaperone GRP78 is known to confer resistance to apoptosis [18]. Calreticulin and GRP78 are most likely involved in the folding of HCV glycoproteins [29], [50]. We and others have now demonstrated that HCV increases GRP78 in HCV-infected cells [27]. In conclusion, we demonstrated that HCV infection caused ER stress, but HCV NS5A confers resistance to ER stress-induced apoptosis. Together, our results reconfirmed that HCV NS5A not only plays a role in HCV-related pathogenesis, but also might be an attractive target of antiviral and antitumor drugs.
